# S4-2 Target group specific policy monitoring for physical activity promotion: development of a new tool in Germany

**DOI:** 10.1093/eurpub/ckad133.019

**Published:** 2023-09-11

**Authors:** Sven Messing, Peter Gelius, Karim Abu-Omar, Franziska Beck, Wolfgang Geidl, Eva Grune, Isabel Marzi, Antonina Tcymbal, Anne Reimers, Klaus Pfeifer

**Affiliations:** Department of Sport Science and Sport, Friedrich-Alexander-Universität Erlangen-Nürnberg; Department of Sport Science and Sport, Friedrich-Alexander-Universität Erlangen-Nürnberg; Department of Sport Science and Sport, Friedrich-Alexander-Universität Erlangen-Nürnberg; Department of Sport Science and Sport, Friedrich-Alexander-Universität Erlangen-Nürnberg; Department of Sport Science and Sport, Friedrich-Alexander-Universität Erlangen-Nürnberg; Department of Sport Science and Sport, Friedrich-Alexander-Universität Erlangen-Nürnberg; Department of Sport Science and Sport, Friedrich-Alexander-Universität Erlangen-Nürnberg; Department of Sport Science and Sport, Friedrich-Alexander-Universität Erlangen-Nürnberg; Department of Sport Science and Sport, Friedrich-Alexander-Universität Erlangen-Nürnberg; Department of Sport Science and Sport, Friedrich-Alexander-Universität Erlangen-Nürnberg

## Abstract

**Purpose:**

While there are several approaches to collect basic information on physical activity (PA) promotion policies, some governments require more in-depth overviews on the situation in their country. In 2021, the German Federal Ministry of Health expressed its interest in collecting detailed data on target group specific PA promotion, as relevant competences in Germany are distributed across a wide range of political levels and sectors. This study describes a new tool developed to reflect German policymakers’ needs for rapid, adaptable, and target group specific monitoring, with a focus on routine national PA promotion practices.

**Methods:**

Based on relevant national and international guidelines, a tool was co-produced by researchers and ministry officials. It includes (1) PA recommendations, (2) national prevalence rates, (3) recommendations for PA promotion, and data on national (4) routine practices, (5) pilot projects and (6) policies. In a pilot study, data were collected for children and adolescents in Germany using desk research, semi-structured interviews and secondary data analysis.

**Results:**

A policy brief and scientific background document were developed. Results showed that 46% of the 4–5-year-olds fulfil WHO recommendations but only 15% of the 11–17-year-olds, and that girls are less active than boys. Currently, no valid data are available on the PA behaviour of 0–3-year-olds in Germany. An overview of routine practices for PA promotion for children and adolescents was compiled, and experts were asked to critically assess their effectiveness, reach and durability. Overall, 339 target group specific projects for PA promotion were found, with 22 classified as examples of good practice. National PA policies for children and adolescents were identified across different sectors and settings.

**Conclusions:**

The new tool provides an added value for PA policy monitoring as it is target group specific, has a unique focus on routine practices in PA promotion, and systematically identifies policy gaps. Starting in 2023, it will serve as a conceptual basis for the development of three additional PA policy briefs tendered by the German government. While developed to specifically suit the German context, it has the potential to be adapted to other countries.

